# 2-Carba-lysophosphatidic acid is a novel β-lysophosphatidic acid analogue with high potential for lysophosphatidic acid receptor activation and autotaxin inhibition

**DOI:** 10.1038/s41598-021-96931-2

**Published:** 2021-08-30

**Authors:** Keiko Fukasawa, Mari Gotoh, Akiharu Uwamizu, Takatsugu Hirokawa, Masaki Ishikawa, Yoshibumi Shimizu, Shinji Yamamoto, Kensuke Iwasa, Keisuke Yoshikawa, Junken Aoki, Kimiko Murakami-Murofushi

**Affiliations:** 1 Ochadai Academic Production, Ochanomizu University, 2-1-1 Ohtsuka, Bunkyo-ku, Tokyo, 112-8610 Japan; 2 Institute for Human Life Innovation, Ochanomizu University, 2-1-1 Ohtsuka, Bunkyo-ku, Tokyo, 112-8610 Japan; 3 Department of Health Chemistry, Graduate School of Pharmaceutical Sciences, The University of Tokyo, 7-3-1 Hongo, Bunkyo-ku, Tokyo, 113-0033 Japan; 4 AMED-LEAP and AMED-CREST, Japan Science and Technology Corporation, 4-1-8 Honcho, Kawaguchi, Saitama 332-0012 Japan; 5 Cellular and Molecular Biotechnology Research Institute, National Institute of Advanced Industrial Science and Technology, 2-4-7 Aomi, Koto-ku, Tokyo, 135-0064 Japan; 6 Transborder Medical Research Center, University of Tsukuba, 1-1-1 Tennodai, Tsukuba, Ibaraki 305-8575 Japan; 7 Division of Biomedical Science, Faculty of Medicine, University of Tsukuba, 1-1-1 Tennodai, Tsukuba, Ibaraki 305-8575 Japan; 8 Clinical Omics Unit, Department of Applied Genomics, Kazusa DNA Research Institute, 2-5-23 Kazusa Kamatari, Kisarazu, Chiba 292-0818 Japan; 9 Department of Pharmacology, Faculty of Medicine, Saitama Medical University, 38 Moro-hongo, Moroyama-machi, Iruma-gun, Saitama, 350-0495 Japan; 10 Present Address: Laboratory of Racing Chemistry, 1731-2 Tsurutamachi Utsunomiya, Tochigi, 320-0851 Japan

**Keywords:** Lipids, Cell signalling

## Abstract

Cyclic phosphatidic acid (cPA) is a naturally occurring phospholipid mediator that, along with its chemically stabilized analogue 2-carba-cyclic phosphatidic acid (2ccPA), induces various biological activities in vitro and in vivo. Although cPA is similar to lysophosphatidic acid (LPA) in structure and synthetic pathway, some of cPA biological functions apparently differ from those reported for LPA. We previously investigated the pharmacokinetic profile of 2ccPA, which was found to be rapidly degraded, especially in acidic conditions, yielding an unidentified compound. Thus, not only cPA but also its degradation compound may contribute to the biological activity of cPA, at least for 2ccPA. In this study, we determined the structure and examined the biological activities of 2-carba-lysophosphatidic acid (2carbaLPA) as a 2ccPA degradation compound, which is a type of β-LPA analogue. Similar to LPA and cPA, 2carbaLPA induced the phosphorylation of the extracellular signal-regulated kinase and showed potent agonism for all known LPA receptors (LPA_1–6_) in the transforming growth factor-α (TGFα) shedding assay, in particular for LPA_3_ and LPA_4_. 2carbaLPA inhibited the lysophospholipase D activity of autotaxin (ATX) in vitro similar to other cPA analogues, such as 2ccPA, 3-carba-cPA, and 3-carba-LPA (α-LPA analogue). Our study shows that 2carbaLPA is a novel β-LPA analogue with high potential for the activation of some LPA receptors and ATX inhibition.

## Introduction

**Figure 1:**
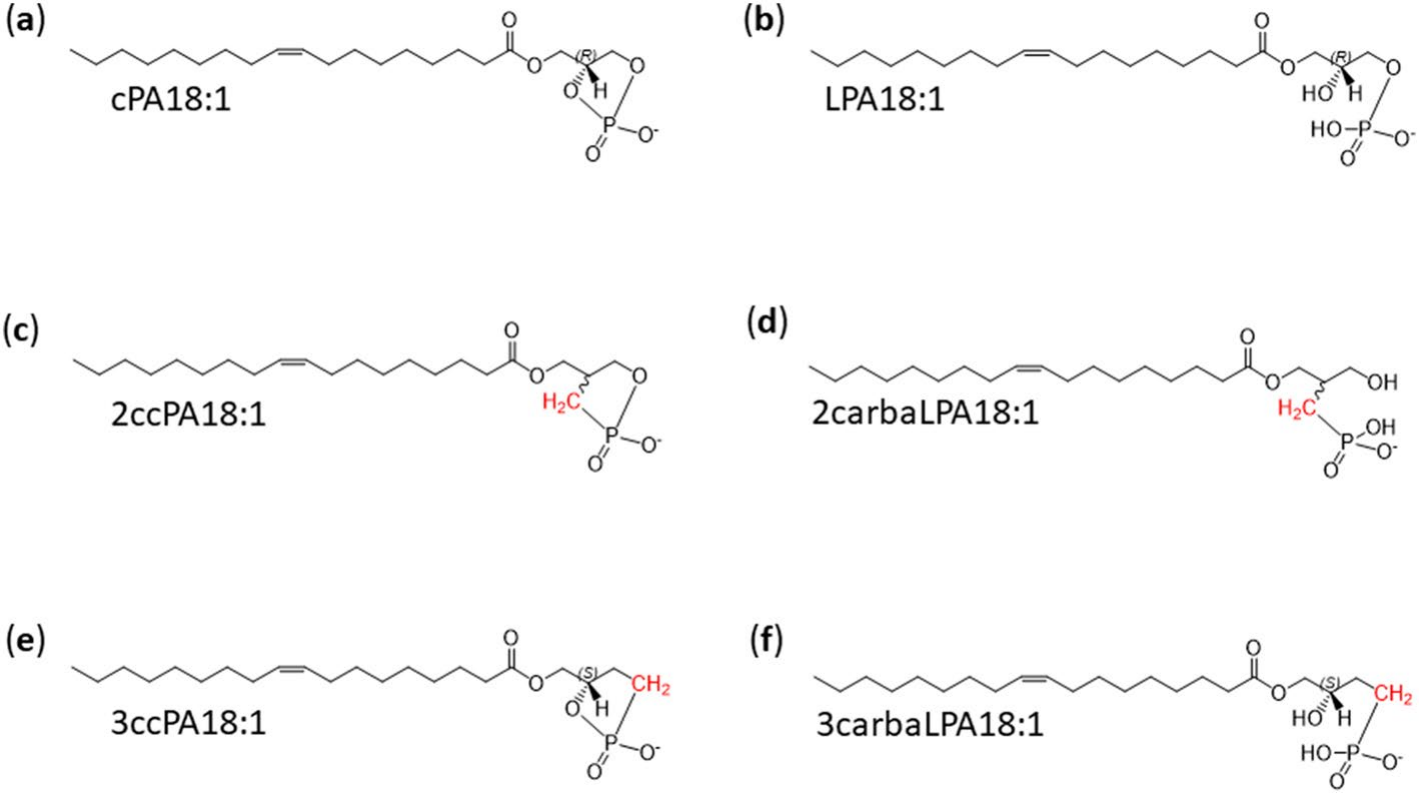
Chemical structure of (**a**) cyclic phosphatidic acid (cPA 18:1), (**b**) lysophosphatidic acid (LPA 18:1), (**c**) 2-carba-cyclic phosphatidic acid (2ccPA 18:1), (**d**) 2-carba-lysophosphatidic acid (2carbaLPA 18:1), (**e**) 3-carba-cyclic phosphatidic acid (3ccPA 18:1), and (**f**) 3-carba-lysophosphatidic acid (3carbaLPA 18:1).

2-Carba-cyclic phosphatidic acid (2ccPA) is a chemically synthesized analogue of cyclic phosphatidic acid (cPA) (Fig. 1)^1^ with promising bioactivity as therapeutic agent. cPA was first isolated from *Physarum polycephalum*, the myxoamoebae of a true slime mould^2^, and was also reported in various natural resources including mammalian tissues and blood^3–5^. cPA contains a cyclic phosphate between *sn*-2 and *sn*-3 of the glycerol backbone, and the replacement of the *sn*-2 oxygen by a methylene group generates the highly stable 2ccPA^4^. Preceding studies have shown that 2ccPA is much potent in inducing several biological activities compared with naturally occurring cPA, which include suppression of lysophospholipase D (lysoPLD) activity of autotaxin (ATX), tumour cell migration^1,6^, delay of neuronal death following transient ischemia^7^, neuropathic pain^8^, and cuprizone-induced demyelination (demonstrated in a multiple sclerosis model)^9^. In mammalian blood, cPA is produced by ATX^10^, which is considered to be a producing enzyme for another bioactive lipid, lysophosphatidic acid (LPA)^11,12^. Interestingly, however, the bioactivities of cPA and 2ccPA are often different from those of LPA^13–15^. To understand the molecular mechanisms of cPA and 2ccPA, it is important to clarify how cPA, 2ccPA, as well as their degradation compounds, interact with the LPA receptor and contribute to their biological function.

To develop 2ccPA as a therapeutic agent, we previously determined its pharmacokinetic profile and observed that 2ccPA is degraded under acidic conditions, resulting in a major degradation compound^16^. In this study, as a continuing investigation to examine the biological activities of 2ccPA, we identified the 2ccPA degradation compound using quadrupole time-of-flight mass spectrometry (QqTOF) and examined its biological activities, including its effect on the phosphorylation of the extracellular signal-regulated kinase (ERK), its activity toward six known LPA receptors (LPA_1–6_), and its inhibitory activity against lysoPLD activity of ATX along with docking simulation with ATX.

## Results and discussion

### Structural characterization of the 2ccPA degradation compound

In a previous study, we found that 2ccPA degraded under acidic conditions and produced a major degradation compound^16^, which was predicted to be a hydrolysate of 2ccPA. We performed exact mass measurements of this degradation compound by direct infusion on QqTOF to characterize its structure. The degradation compound was purified by extraction from one spot on a thin layer chromatography (TLC) plate and its purity was confirmed by QqTOF along with liquid chromatography coupled to triple quadrupole mass spectrometry (LC-QqQ).

**Figure 2:**
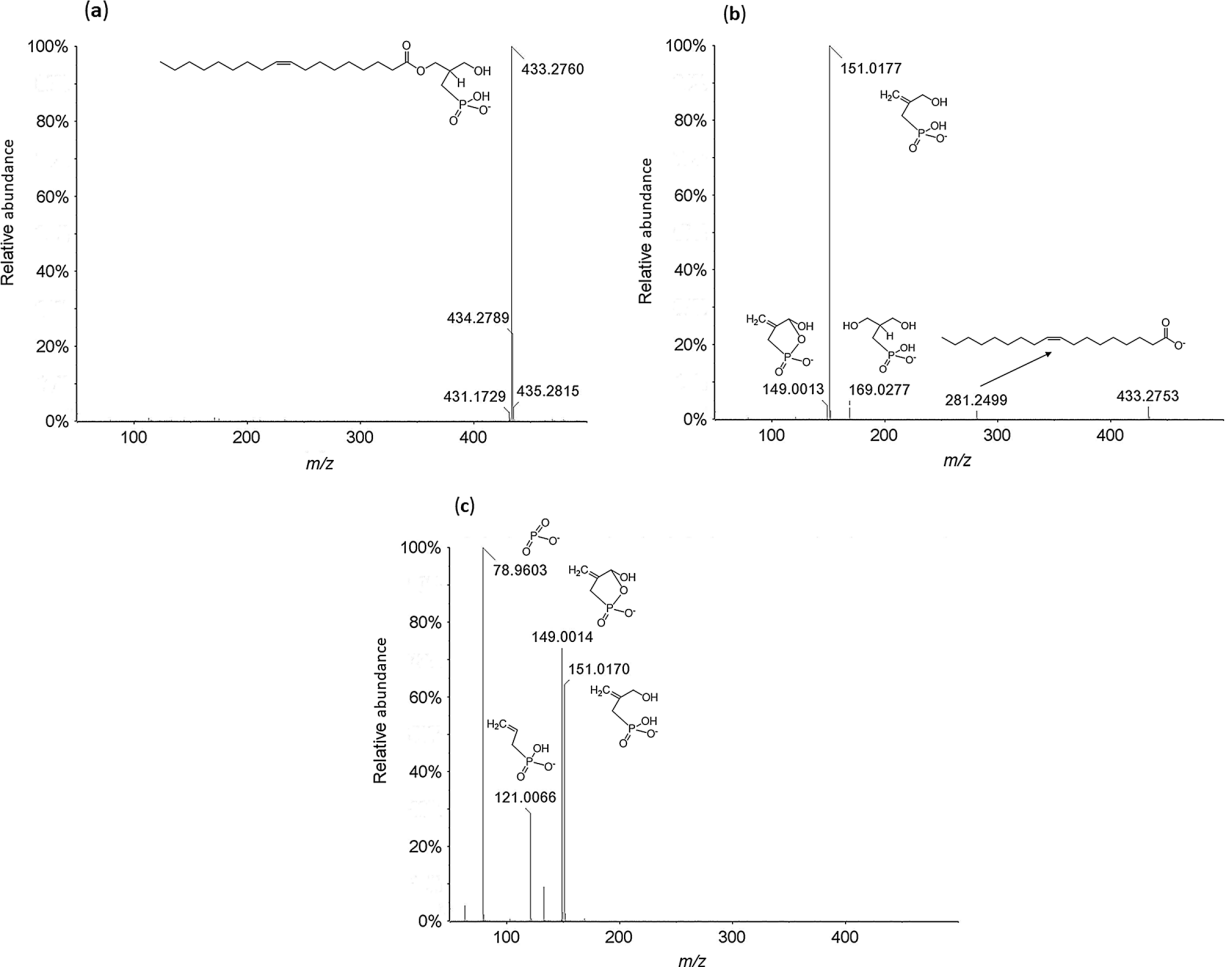
Full mass negative ion scan and proposed chemical structure of (**a**) 2-carba-cyclic phosphatidic acid (2ccPA) degradation compound determined by quadrupole time-of-flight mass spectrometry (QqTOF). (**b**, **c**) Product ion analysis of 2ccPA degradation compound at *m*/*z* 433.2760 performed by QqTOF using a peak width setting of 1.4 Th, by selection of the molecular ion in the first quadrupole and collision activation in the second quadrupole with a collision energy of (**b**) − 30 and (**c**) − 75 eV. Proposed structures of the product ions observed in the tandem mass spectrometry spectra are shown. Background subtraction was performed using the PeakView software version 1.2 (Sciex).

Negative ion full mass spectrometry (MS) scan analysis showed that the predominant *m/z* value of the 2ccPA degradation compound was 433.2760 (Fig. 2a). Tandem MS spectra of this degradation compound at collision energy (CE) − 30 and − 75 eV are illustrated in Fig. 2b,c, respectively. At CE − 30 eV, the fragment ions were detected at *m/z* 149.0013, 151.0177, 169.0277, and 281.2499 (Fig. 2b). These results suggested that the structure of the degradation compound was open at the position where the oxygen in the phosphate group was present owing to the strong acidic treatment of 2ccPA (Fig. 2a). In the presumed structure, the mass measurement error and formula of the precursor ion were 9.5 ppm and C_22_H_42_O_6_P^−^, respectively. The mass measurement errors for the product ions at *m/z* 149.0013, 151.0177, 169.0277, and 281.2499 were 6.0, 11.3, 6.5, and 6.4 ppm (formula: C_4_H_6_O_4_P^−^, C_4_H_8_O_4_P^−^, C_4_H_10_O_5_P^−^, and C_18_H_33_O_2_^−^), respectively. Based on this mass accuracy, we assigned these fragment ions to structural features derived from the 2ccPA hydrolysis product (Fig. 2b). In high-energy collisions (CE: − 75), smaller fragment ions (*m/z*: 78.9603 and 121.0066) were generated by further fragmentation of the polar head group and glycerol backbone, respectively (Fig. 2c). These results indicate that the degradation compound opened the cyclic structure of 2ccPA at the *sn*-3 oxygen while the phosphate group remained attached to the *sn*-2 methylene (Fig. 1d). We named this 2ccPA degradation compound as 2-carba-lysophosphatidic acid (2carbaLPA). Similarly, 3-carba-cyclic phosphatidic acid (3ccPA, Fig. 1e) was converted to the corresponding 3-carba-lysophosphatidic acid (3carbaLPA, Fig. 1f) under the same acidic condition, suggesting that the cyclic structure of 3ccPA opened at the *sn*-2 oxygen, leaving the remaining phosphate group attached to the *sn*-3 methylene (Supplementary Fig. S1). 3carbaLPA was previously synthesized and reported by Jiang et al.^17^ as one of the α-substituted phosphonate analogues of LPA. However, to our knowledge, this is the first study to show that 2carbaLPA, which is a β-LPA analogue bearing a phosphate group attached at the *sn*-2 position of the glycerol backbone, could be converted from 2ccPA.

### 2carbaLPA induces ERK phosphorylation via LPA_1_, LPA_2_, and/or LPA_3_

**Figure 3:**
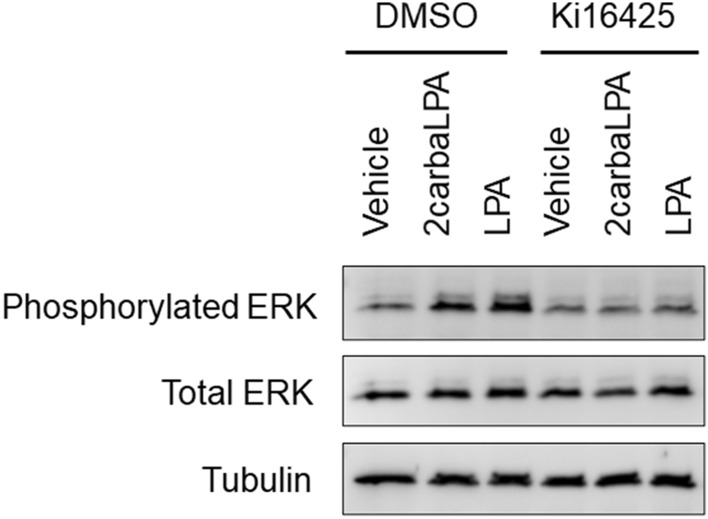
Effects of 2-carba-lysophosphatidic acid (2carbaLPA) on the phosphorylation of the extracellular signal-regulated kinase (ERK) protein. HeLa cells were preincubated with 10 µM Ki16425 for 30 min at 37 °C and then incubated with 10 µM 2carbaLPA, lysophosphatidic acid (LPA) 18:1, or vehicle for 5 min at 37 °C. Protein levels of phosphorylated ERK, total ERK, and α-tubulin were determined by western blot analysis. The full-length blots are shown in the Supplementary Fig. S2.

ERK is an important regulator of various biological functions, such as cell proliferation, motility, and apoptosis^18^, and was shown to be activated (phosphorylated) by LPA signalling. Analysis of ERK phosphorylation in HeLa cells revealed that 2carbaLPA activated ERK (Fig. 3, Supplementary Fig. S2), an effect that was inhibited by Ki16425, a LPA_1–3_ antagonist. These results indicate that 2carbaLPA stimulates ERK via the LPA_1_, LPA_2_, and/or LPA_3_ receptors. Hence, 2carbaLPA may perform several biological functions via ERK activation.

### 2carbaLPA is a potent agonist of LPA receptors

**Figure 4:**
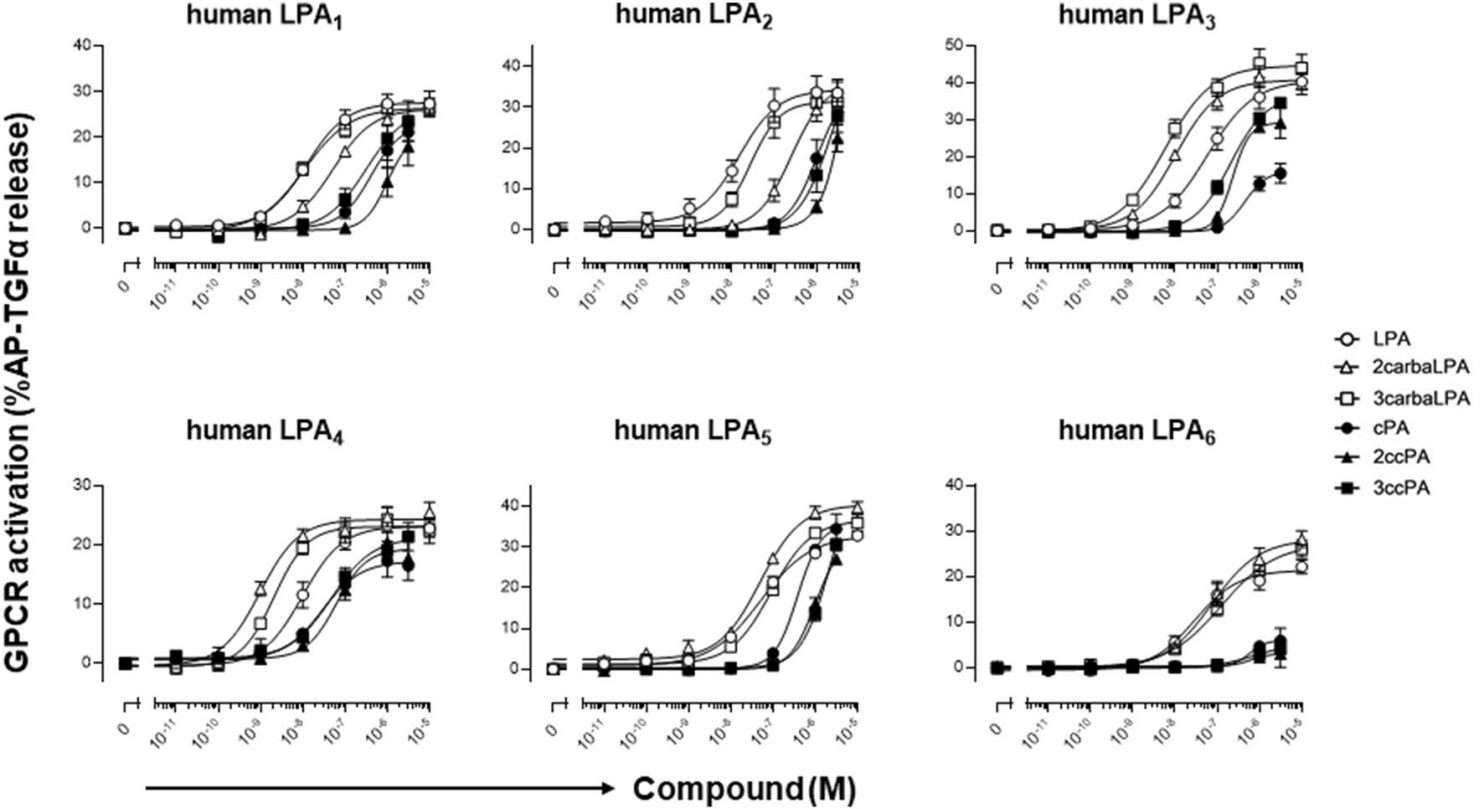
Agonistic activity of 2-carba-lysophosphatidic acid (2carbaLPA) and cyclic phosphatidic acid (cPA) analogues toward six lysophosphatidic acid (LPA) receptors. 293A cells expressing LPA receptors were stimulated with the indicated concentration of LPA, 2carbaLPA, and cPA analogues. Receptor-specific alkaline phosphatase-tagged transforming growth factor-α release was calculated by subtracting the responses in the empty vector-transfected cells from those in the receptor-expressing cells. Data are represented as standard error of the mean of three independent replicates.

Next, we performed the transforming growth factor (TGF)-α-shedding assay in 293A cells to evaluate the potency of 2carbaLPA (β-LPA analogue), as well as 3carbaLPA (α-LPA analogue) and cPA analogues, to activate LPA_1–6_. As shown in Fig. 4, 2carbaLPA was found to be a potent agonist for all six LPA receptors, especially for LPA_3_ and LPA_4_. 2carbaLPA concentrations inducing the half-maximal response (EC_50_) were of 54.8, 256.4, 11.6, 1.0, 50.2, and 90.9 nM for LPA_1_, LPA_2_, LPA_3_, LPA_4,_ LPA_5_, and LPA_6_, respectively (Table 1). 3carbaLPA also exerted a similar potent agonistic activity toward LPA_1–6_. Jiang et al.^17^ reported that 3carbaLPA exhibits agonistic activity toward LPA_1–4_ in the Ca^2+^ mobilization assay. Contrasting with our results, they^17^ showed that the activity of 3carbaLPA for LPA_1-4_ is much weaker than that of LPA; particularly toward LPA_3_ and LPA_4_ for which was more than 10-times weaker than that of LPA. These different results could be attributed to the type of assay. The one of the differences between TGFα-shedding assay and Ca^2+^ mobilization assay is incubation period applied. In the Ca^2+^ mobilization assay, LPA receptor-expressing RH7777 and CHO cells were used, along with less than 2 min incubation time with the compounds, whereas the incubation period in the TGFα-shedding assay was 1 h, and the stability of the compounds may affect the results. We measured the stability of the compounds during 1 h, and revealed that 3carbaLPA and the other compounds were mostly stable, except LPA which concentration was reduced by up to 30% (with approximately 70% remaining, Supplementary Fig. S3). Therefore, the difference between the findings of our study and the previous study on the potency of 3carbaLPA toward LPA receptors may be attributed to the incubation period.

**Table 1:** Agonistic activity of 2carbaLPA and cPA analogues toward the six human LPA receptors.

Compounds	EC_50_ (LogEC_50_ ± SEM) [E_max_ ± SEM]					
	hLPA_1_	hLPA_2_	hLPA_3_	hLPA_4_	hLPA_5_	hLPA_6_
LPA 18:1	11.4 nM (− 7.94 ± 0.09) [26.9 ± 2.3%]	14.8 nM (− 7.83 ± 0.08) [32.9 ± 3.1%]	61.1 nM (− 7.21 ± 0.15) [40.8 ± 2.1%]	10.4 nM (− 7.98 ± 0.13) [24.8 ± 0.8%]	52.8 nM (− 7.28 ± 0.11) [32.2 ± 1.9%]	34.0 nM (− 7.47 ± 0.14) [22.6 ± 1.3%]
2carbaLPA 18:1	54.8 nM (− 7.26 ± 0.03) [27.0 ± 2.2%]	256.4 nM (− 6.59 ± 0.09) [35.8 ± 2.1%]	11.6 nM (− 7.94 ± 0.08) [42.7 ± 3.2%]	1.0 nM (− 9.02 ± 0.10) [24.9 ± 1.5%]	50.2 nM (− 7.30 ± 0.04) [38.3 ± 1.8%]	90.9 nM (− 7.04 ± 0.21) [29.3 ± 1.2%]
3carba LPA 18:1	11.0 nM (− 7.96 ± 0.12) [26.9 ± 1.9%]	28.3 nM (− 7.55 ± 0.07) [30.7 ± 2.8%]	5.6 nM (− 8.25 ± 0.12) [44.3 ± 4.0%]	1.9 nM (− 8.72 ± 0.10) [23.5 ± 1.5%]	89.3 nM (− 7.05 ± 0.14) [35.5 ± 1.8%]	132.2 nM (− 6.88 ± 0.05) [27.1 ± 2.0%]
cPA 18:1	459.6 nM (− 6.34 ± 0.26) [24.1 ± 1.1%]	1067.4 nM (− 5.97 ± 0.08) [35.9 ± 4.3%]	439.5 nM (− 6.36 ± 0.11) [16.8 ± 2.2%]	32.8 nM (− 7.48 ± 0.11) [17.2 ± 0.8%]	354.3 nM (− 6.45 ± 0.12) [35.6 ± 4.1%]	> 3 µM (> − 5.5) NA
2ccPA 18:1	996.9 nM (− 6.00 ± 0.04) [20.6 ± 4.9%]	> 3 µM (> − 5.5) NA	286.4 nM (− 6.54 ± 2.67) [30.8 ± 4.0%]	71.9 nM (− 7.14 ± 0.19) [19.6 ± 2.7%]	972.7 nM (− 6.01 ± 0.06) [31.7 ± 0.1%]	> 3 µM (> − 5.5) NA
3ccPA 18:1	369.5 nM (− 6.43 ± 0.25) [26.9 ± 4.2%]	2596.2 nM (− 5.59 ± 0.17) [53.2 ± 13.5%]	216. 8 nM (− 6.66 ± 0.12) [37.4 ± 1.6%]	92.3 nM (− 7.03 ± 0.28) [23.3 ± 2.6%]	2233.6 nM (− 5.65 ± 0.16) [51.2 ± 12.9%]	> 3 µM (> − 5.5) NA

Data are represented as the mean of three independent replicates.

*2carbaLPA* 2-carba-lysophosphatidic acid, *2ccPA* 2-carba-cyclic phosphatidic acid, *3carbaLPA* 3-carba-lysophosphatidic acid, *3ccPA* 3-carba-cyclic phosphatidic acid, *cPA* cyclic phosphatidic acid, *hLPA*_*1–6*_ human LPA receptors, *NA* not available because of very low activity.

Multiple LPA receptors are expressed on most animal cells and act cooperatively. LPA receptors are simultaneously activated by LPA and the signals are mediated via cross-talk by intracellular signalling, which determines the appropriate cellular behaviour. For example, LPA_1_ and LPA_5_ receptors were reported to perform disparate functions in tumour cells^19^. Therefore, the levels of each LPA receptor in individual cells, their affinity for the ligand, and efficacy of the ligand are important factors for multiple LPA receptor signalling. With respect to affinity for LPA receptors, LPA_2_ is known to be activated at 10-times lower concentrations than that observed for LPA_1_, in Ca^2+^ mobilization assays^1^. Therefore, even if the expression of LPA_1_ is higher than that of LPA_2_, the input of LPA_2_ signalling may be prioritized. Moreover, under natural conditions, there are several LPA species present, in which the acyl chains affect the affinity for the LPA receptors^20^. The process underlying the determination of a comprehensive priority order considering multiple LPA receptor signalling patterns in individual cells is highly complex. Several tools have been used to elucidate this process, including receptor-specific agonists, antagonists, inhibitors, and LPA receptor knockout models. It is noteworthy that 2carbaLPA and 3carbaLPA exhibit significantly higher agonistic potential than LPA toward LPA_3_ and LPA_4_, in TGFα-shedding assays. LPA_3_ contributes to ovarian cancer malignancy^21^, and pancreatic cancer progression and migration^22^. Conversely, LPA_4_ performs contrasting actions, such as regulation of cellular motility in non-malignant cells^23^. As envisaged earlier, cPA and LPA may elicit bioactivities through different cell signalling pathways^13–15^; therefore, the cell signalling pathways of 2carbaLPA and 3carbaLPA should be clarified and their biological functions should be investigated.

### 2carbaLPA strongly inhibits the lysophospholipase D activity of ATX

LPA is known to act as a crucial lipid mediator that regulates various cellular functions, such as proliferation, migration, and differentiation^24^. ATX was identified as an LPA producing extracellular enzyme in mammalian blood^11,12^; thus, multiple attempts have been made to develop ATX inhibitors^25,26^. We have found that cPA, a naturally occurring LPA analogue, inhibits ATX^1^. Moreover, we have synthesized effective ATX inhibitors, such as 2ccPA and 3ccPA^1^. In this study, we investigated the effectiveness of 2carbaLPA on the lysoPLD activity of ATX, in addition to those of other cPA analogues, using two types of assays—detection of LPC cleavage using choline oxidase and detection of synthesized LPA using LC-QqQ.

**Figure 5:**
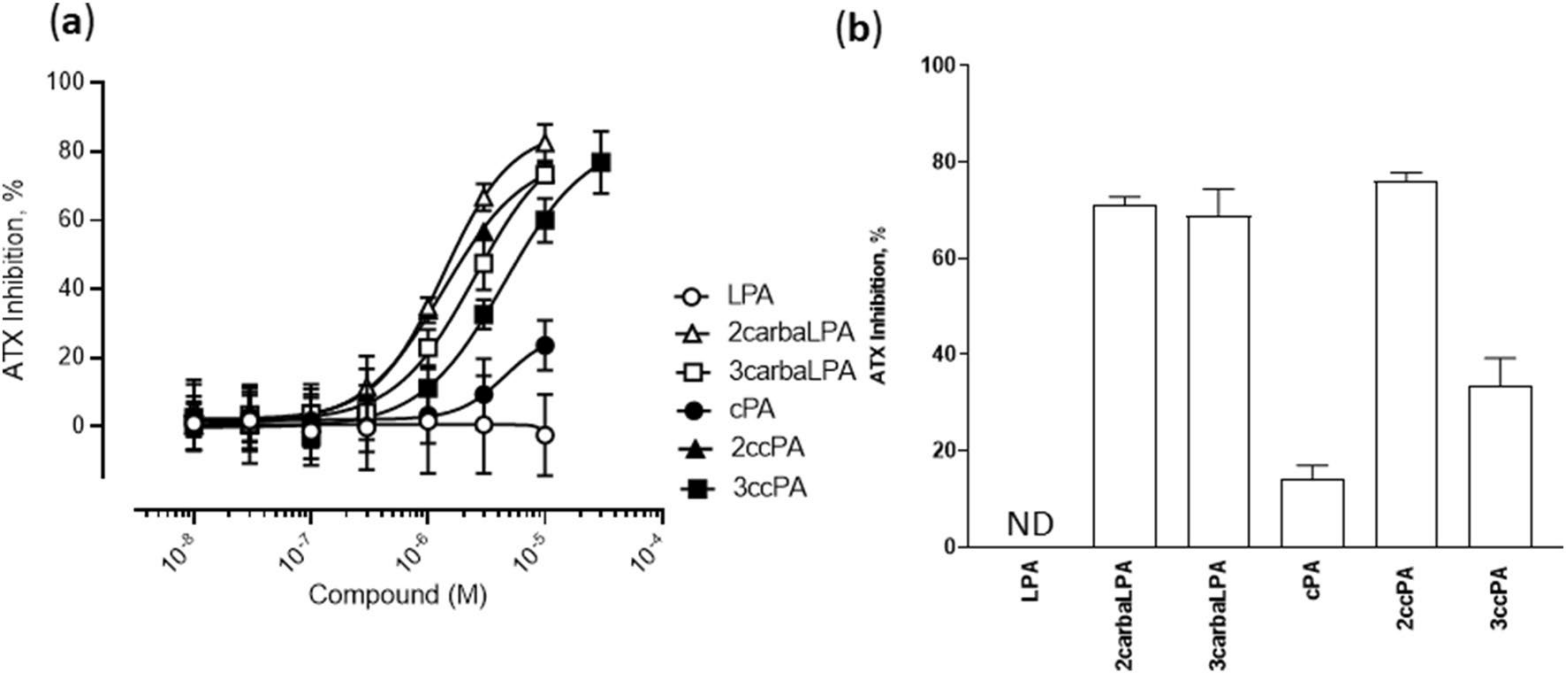
Inhibition of lysophospholipase D activity of autotaxin (ATX) by cyclic phosphatidic acid (cPA) analogues. (**a**) Concentration–response curves for the inhibition of ATX activity as measured by the hydration of the substrate lysophosphatidylcholine (LPC) using choline oxidase and peroxidase. LPC 16:0 (final concentration: 300 µM, above its critical micellar concentration [CMC]), human recombinant ATX (final concentration: 50 nM), and various concentrations of cPA analogues were used for incubation with 4-aminoantipyrine, TOOS (final concentration: 0.05% (w/v) each), choline oxidase and peroxidase (final concentration: 10 U/mL each) in the ATX assay buffer. The absorbance at a wavelength of 555 nm was measured at 0 and 2 h incubation at 37 °C. Inhibition was calculated as percentage (%) of reduction in absorbance compared with absorbance in the vehicle control (without cPA analogues) after subtraction of the value measured at time 0. Data are represented as mean ± standard deviation (n = 3–4 samples). (**b**) Inhibition of ATX activity measured by lysophosphatidic acid (LPA 16:0) production via liquid chromatography coupled to triple quadrupole mass spectrometry (LC-QqQ). The substrate LPC 16:0 (final concentration: 300 µM) was incubated with human recombinant ATX (final concentration: 50 nM) and cPA analogues (final concentration: 10 μM) for 2 h at 37 °C. The ATX product, LPA 16:0, was quantified by LC-QqQ. Inhibition was calculated as percentage (%) of reduction in LPA production compared with LPA production in the vehicle control (without cPA analogues). Data are represented as mean ± standard deviation (n = 5–6 samples).

In the choline oxidase assay used for detection of the choline moiety produced from LPC, which is an endogenous substrate of ATX, 2carbaLPA inhibited the lysoPLD activity of ATX in a dose-dependent manner, similar to 2ccPA and 3carbaLPA (Fig. 5a). 3ccPA exhibited lower inhibitory activity than the other analogues, except for LPA and cPA. This finding was consistent with those of our previous study, in which 3ccPA showed lower inhibitory activity than 2ccPA^1,27^. Notably, cPA exhibited significantly lower inhibitory activity and LPA exhibited almost no inhibitory activity under our present conditions.

By measuring the reaction product of ATX/LyoPLD using LC-QqQ, i.e., LPA, we observed that 2carbaLPA and 3carbaLPA showed similar ATX inhibitory activities (Fig. 5b). cPA was found to be a poor ATX inhibitor. Notably, the activity exhibited by 3ccPA was also weak; however, that of 3carbaLPA, the hydrolysed compound of 3ccPA, was significantly stronger than that of 3ccPA.

**Figure 6:**
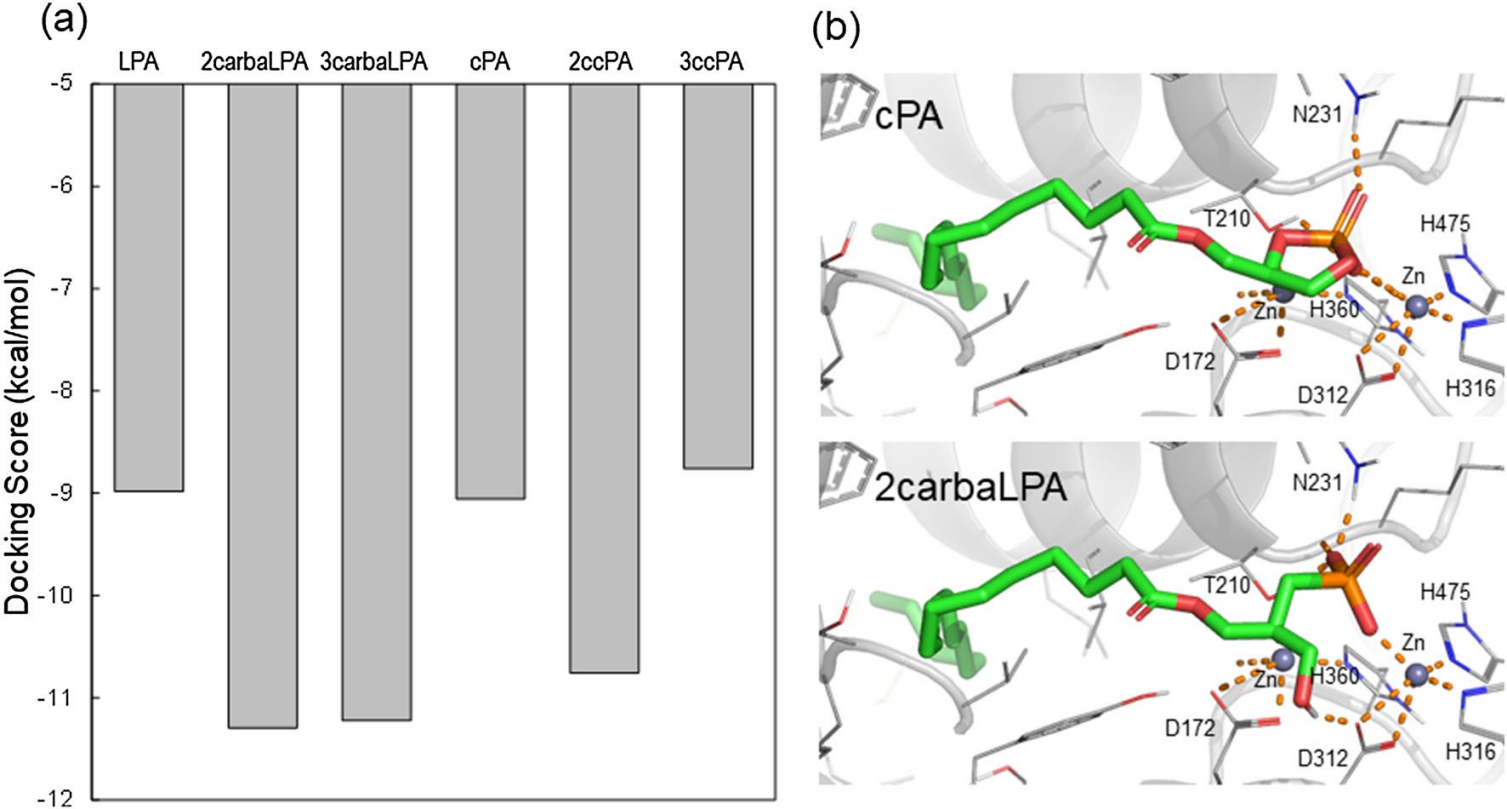
Docking simulation of cyclic phosphatidic acid (cPA) analogues and autotaxin (ATX). (**a**) Grey colour indicates the docking score of cPA analogues. (**b**) Proposed models for cPA (top) and 2-carba-lysophosphatidic acid (2carbaLPA, bottom) binding to ATX (diagram representation coloured in grey) from docking simulation. ATX residues around the compounds within 4 Å and compounds are shown in grey line and green stick representation, respectively. Polar interaction between ATX and the compounds are depicted by orange dots. The structural models in the figures were depicted using PyMOL version 2.4 software (Schrödinger, LLC).

Furthermore, we performed docking simulation to extract structure activity relationship (SAR) regarding complexes formed by cPA analogues and ATX. The cPA analogues docked into the active site of ATX in a similar manner as LPA, including coordinating oxygen atom of the phosphate group with the Zn^2+^ ion and the hydrophobic interaction of the acyl-chain. The conformational changes of the glycerol backbone moiety into the active site of ATX determined based on docking simulation are an important functional property of cPA analogues. It was also observed a correlation tendency between the docking score (Fig. 6a) and inhibition of ATX activity (Fig. 5a,b). The difference in inhibitory activity against ATX between cPA and 2carbaLPA was further examined by docking models, which revealed that the hydroxy group of 2carbaLPA forms an additional hydrogen bond with the side chain carboxyl group of Asp312 at the LPA binding pocket of ATX (Fig. 6b). These results indicate that 2carbaLPA may have a higher binding affinity than cPA and, as a result, 2carbaLPA becomes a potent inhibitor of ATX.

There are numerous methods to evaluate ATX inhibitory activity in vitro, using endogenous substrates and artificial substrates^28^. For evaluating ATX inhibition, choline oxidase assay or detection of LPA using LC-QqQ, both of which use the endogenous substrate LPC, is compatible method. LPA was reported by Van Meeteren et al*.* to exhibit product inhibition against ATX in experiments using artificial substrate, such as labelled LPC, TMP *p*-nitrophenyl (*p*NP-TMP), bis(*p*-nitrophenyl) phosphate (bis-pNPP), or fluorescence resonance energy transfer-based phosphodiesterase sensor^29^. However, we again observed that LPA does not inhibit ATX in an experiment using intact LPC as substrate. This was consistent with the results reported by Stein et al.^30^ and Salgado-Polo et al.^31^ using the choline oxidase assay. The latter group recently proposed LPA as a positive allosteric modulator of ATX, as evidenced by a detailed kinetic study using choline oxidase assay among other methods^31,32^. Thus far, several artificial substrates of ATX have been developed to evaluate ATX activity. However, owing to the broad substrate specificity of ATX, the substrates should be selected carefully for each study to avoid misleading results.

### A new enzymatic activity of ATX producing α-LPA and β-LPA

In the LC-QqQ assay of ATX activity with cPA analogues, we found that cPA or 2ccPA concentration itself decreased during incubation with LPC and human recombinant ATX. When we incubated only cPA or 2ccPA with ATX, its concentration decreased, whereas concentration of LPA or 2carbaLPA increased in a time-dependent manner (Supplementary Fig. S4a,b). This indicates that ATX hydrolyses cPA to LPA and 2ccPA to 2carbaLPA. Similarly, 3ccPA was also hydrolysed to 3carbaLPA by ATX in vitro (Supplementary Fig. S4c). In a previous study^16^, we administered 2ccPA in an entero-soluble capsule to investigate the pharmacokinetic properties of 2ccPA in rat plasma. We observed that 2carbaLPA was detectable in the plasma after administration of 2ccPA (Supplementary Fig. S5); thus, 2ccPA may be hydrolysed by ATX in vivo. Therefore, 2ccPA may hold several biological functions and 2carbaLPA might be the active form after ATX-hydrolysis.

**Figure 7:**
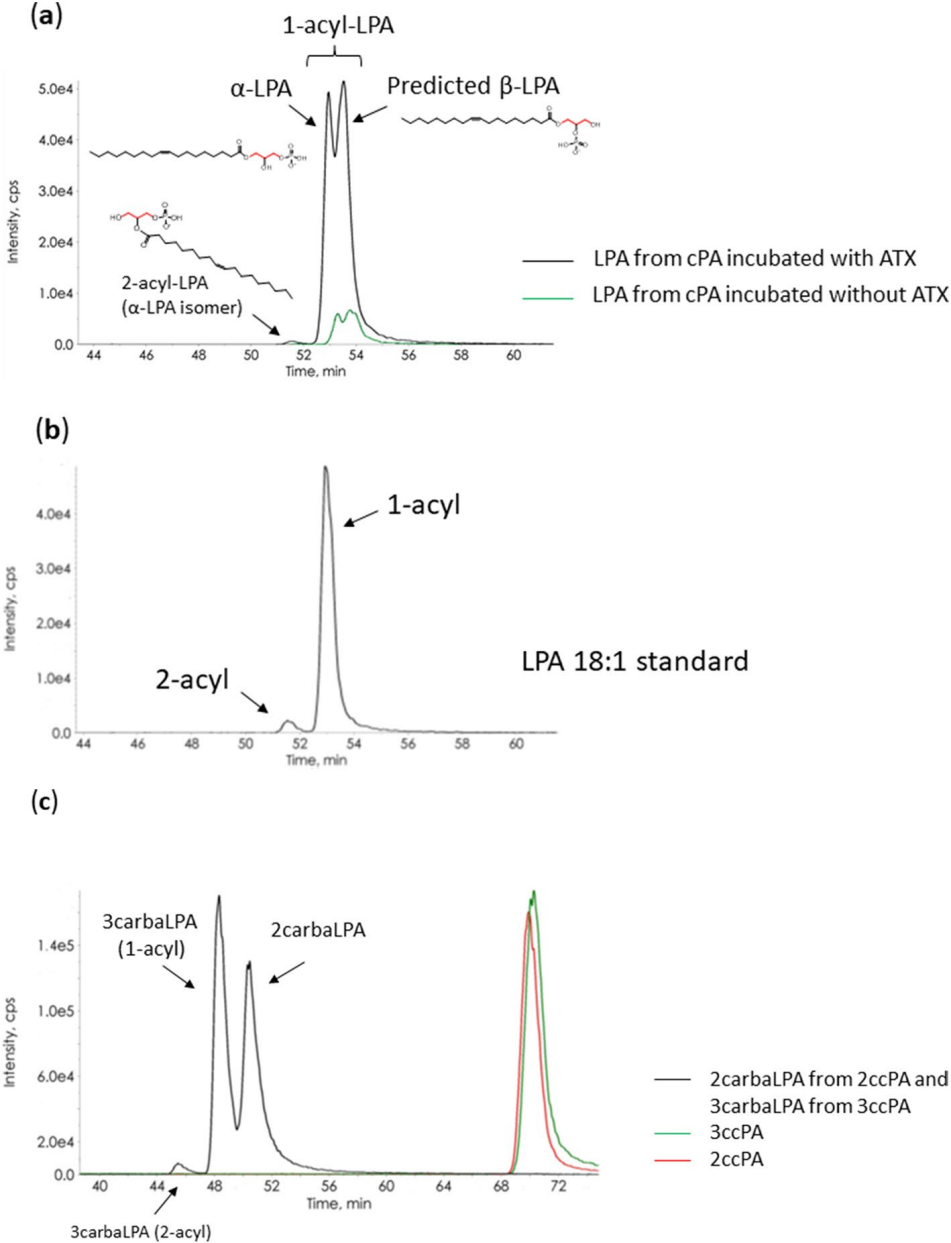
Chromatogram of (**a**) lysophosphatidic acid (LPA) 18:1 produced from cyclic phosphatidic acid (cPA) 18:1, (**b**) standard LPA 18:1 shown for comparison, and (**c**) 2-carba-lysophosphatidic acid (2carbaLPA) and 3-carba-lysophosphatidic acid (3carbaLPA) produced from 2-carba-cyclic phosphatidic acid (2ccPA) and 3-carba-cyclic phosphatidic acid (3ccPA), respectively, after analysis via liquid chromatography coupled to triple quadrupole mass spectrometry (LC-QqQ). The simplified chemical structure of α-LPA (1-acyl), α-LPA isomer (2-acyl), and predicted β-LPA are shown. (**a**) cPA 18:1 (final concentration: 10 μM) and (**c**) 2ccPA 18:1 plus 3ccPA 18:1 mixture (final concentration: 10 μM each) as substrates were incubated, with or without (**a** only) human recombinant autotaxin (ATX, final concentration: 50 nM), for 2 h at 37 °C. LC-QqQ conditions are described in the “Materials and methods” section.

Using 2ccPA and 3ccPA, we clearly showed that ATX could cleave not only the O-P bond in C-O-P at the *sn*-2 position of 3ccPA, but also at the *sn*-3 position of 2ccPA. Indeed, when cPA was incubated with ATX, three types of LPA compounds were formed (Fig. 7a): LPA, which contains a phosphate group attached at the *sn*-3 position of the glycerol backbone (referred to as α-LPA), and its acyl chain readily migrates between the *sn*-1 (1-acyl LPA) and *sn*-2 positions (2-acyl LPA)^33^, as shown in Fig. 7b. We predicted that the additional peak was that of LPA with a phosphate group attached at the *sn*-2 position of the glycerol backbone (β-LPA form). Moreover, we assumed that it could also cleave the O-P bond in C-O-P bond at the *sn*-3 position in LPA and could detach the phosphate group from the glycerol backbone to produce monoacylglycerol. However, we could not detect monoacylglycerol using LC-QqQ after LPA was incubated with ATX (data not shown).

To compare the substrate specificities of ATX for 2ccPA and 3ccPA, we incubated equal concentrations of 2ccPA and 3ccPA with ATX. Hydrolysed compounds, 2carbaLPA and 3carbaLPA, were detected at almost equal levels (Fig. 7c), suggesting that ATX could equally cleave the O–P bond in C–O–P at the *sn-*2 and *sn*-3 positions, which was consistent with the finding that ATX could produce almost the same amount of α-LPA and β-LPA from cPA (Fig. 7a). Thus far, β-LPA has not been detected in vivo, possibly due to the low concentration of cPA (approximately 5–10 nM of each cPA species is present in rat serum)^5^. However, it is possible that there exists a biological pathway for β-LPA synthesis involving the in vivo production of cPA from LPC. Therefore, we are currently isolating β-LPA from cPA and investigating the biological function of β-LPA in another study. Lysophospholipids that have a phosphate group attached at the *sn*-2 position of the glycerol backbone are not detected naturally, but they have been synthesized chemically for testing biological activity (lysophophatidylserine derivatives)^34^. 2carbaLPA is a practical tool that can be used as a model of β-LPA to perform conformational analysis and to evaluate biological activities.

## Conclusions

Using QqTOF, we characterised 2carbaLPA, the degradation compound of 2ccPA, which was generated under acidic conditions, such as those in the stomach. As an analogue of β-LPA, 2carbaLPA phosphorylated the ERK protein and exhibited agonistic activity toward six LPA receptors; particularly, it exhibited higher activity than that observed for LPA toward LPA_3_ and LPA_4_. 2carbaLPA was shown to inhibit the lysoPLD activity of ATX by approximately 80%, which was similar to that observed for 2ccPA and 3carbaLPA, and greater than that observed for 3ccPA (30%) and cPA (10%). From the docking score with ATX, 2carbaLPA was also estimated as most highly active in cPA analogues.

These findings will be useful for understanding the biological mechanism of 2ccPA action, which would influence its application as a therapeutic agent and also improve our knowledge on the ATX-LPA receptor signalling.

## Materials and methods

### Reagents

Human recombinant ATX [specific activity: 5.40 or 3.13 U/mg (batch number 0530156–1 or 0584094, respectively)] was purchased from Cayman Chemical (Ann Arbor, MI, USA). 1-Oleoyl-LPA (LPA 18:1) and LPC 16:0 were purchased from Sigma-Aldrich (St. Louis, MO, USA) or Avanti Polar Lipids (Alabaster, AL, USA). 1-Oleoyl-cPA (cPA 18:1) was purchased from Avanti Polar Lipids. The purity of cPA obtained from Avanti Polar Lipids, which is frequently highly degraded or contaminated with 1-oleoyl-LPA (up to 98%), is usually confirmed using QqTOF. In this study, we used highly pure cPA, purity was confirmed to be above 97%. Additionally, 2ccPA was also prepared as per previously described^4^.

3ccPA for TGFα-shedding assay and preparation of the degradation compound, was generously provided by Prof. Susumu Kobayashi (Tokyo University of Science). For other experiments 3ccPA was purchased from Echelon Biosciences (Salt Lake City, UT, USA). *N*-Ethyl-*N*-(2-hydroxy-3-sulfopropyl)-3-methylaniline, sodium salt, dihydrate (TOOS) was purchased from Dojindo (Kumamoto, Japan). Horseradish peroxidase was purchased from Toyobo Bio (Osaka, Japan). Choline oxidase isolated from *Arthrobacter globiformis* was kindly provided by Imamura Enzyme Technologies Corp. (Shizuoka, Japan). Acidic methanol (pH 4.0) was prepared as per previously described^35^.

### Purification of 2ccPA and 3ccPA degradation compounds

2ccPA was dissolved in artificial gastric juice (2 g NaCl and 12 M HCl 7 mL/1000 mL water at pH 1.2) at a final concentration of 1 mg/mL and incubated for 2 h at 37 °C. Lipid fraction was extracted by 4 volumes of chloroform/methanol (2:1, v/v) and separated by TLC as per previously reported^16^. Extraction from the silica powder of TLC was performed by addition of 8 mL of chloroform/methanol (1:9, v/v) and it was repeated three times. After filtration (0.2 μm Captiva Premium syringe PTFE filter, Agilent Technologies, Santa Clara, CA, USA), the sample was diluted 100-fold with 5 mM ammonium formate in methanol/water (95:5, v/v) and subjected to QqTOF. The 3ccPA degradation compound was also prepared using the same method. The purity of the degradation compound was confirmed to be more than 95% by TLC, QqTOF, and LC-QqQ. Quantification was performed by weighing the purified sample after dried more than 24 h in the desiccator equipped with a vacuum pump.

### QqTOF analysis

Structural characterisation of the 2ccPA degradation compound was performed using a Triple TOF4600 (Sciex, Framingham, MA, USA) mass spectrometer equipped with a DuoSpray ion source. The source conditions were as follow (arbitrary units unless otherwise specified): temperature 0 °C, curtain gas 20, source gas 1 and source gas 2 at 15 and 0, respectively, and ion spray voltage at − 4.5 kV. The declustering potential was − 100 V. Full scan mass spectral data were acquired from *m/z* 50 to 500 within an accumulation time of 250 ms, and CE of − 10 eV. Product ion scan was performed from *m/z* 50 to 500 at precursor *m/z* 433.3 (accumulation time 100 ms, CE − 30 and − 75 eV). Mass calibration was performed daily prior to commencement of the analysis by using an APCI negative calibration solution (Sciex). Data processing was performed using the Sciex Analyst TF software version 1.6 and the Sciex PeakView software version 1.2.

### Western blot analysis of phosphorylated ERK

HeLa cells were obtained from RIKEN RBC Cell Bank (Tsukuba, Japan). The serum-starved cells were preincubated with 10 µM Ki16425 or DMSO for 30 min, and incubated with 10 µM 2carbaLPA or LPA 18:1 in 0.1% fatty acid-free bovine serum albumin (BSA, Sigma-Aldrich)/phosphate-buffered saline (PBS) solution or vehicle (0.1% BSA/PBS) for 5 min at 37 °C. Cells were harvested using 0.125 M Tris–HCl (pH 6.8) supplemented with 4% sodium dodecyl sulphate (SDS), 20% glycerol, 10% 2-mercaptoethanol, and 0.01% bromophenol blue. Proteins were separated by SDS–polyacrylamide gel electrophoresis, and the proteins were transferred onto an Immobilon-P Transfer Membrane (Millipore, Burlington, MA, USA). The membrane was incubated with primary antibodies: anti-phospho-ERK (pERK), anti-ERK, and anti-tubulin (all at 1:1,000 dilution, Cell Signaling Technology, Danvers, MA, USA). Next, after washing, the membrane was treated with horseradish peroxidase-conjugated anti-rabbit IgG (1:1000 dilution; Cell Signaling Technology). Immunodetection was performed using an enhanced chemiluminescence system (EzWestLumi plus, ATTO Corporation, Tokyo, Japan) and ImageQuant LAS 4000 (GE healthcare, Buckinghamshire, UK).

### TGFα-shedding assay

293A cells (Life Technologies, Tokyo, Japan) were cultured in DMEM (Nissui Pharmaceutical, Tokyo, Japan) supplemented with 10% foetal calf serum (Gibco, Waltham, MA, USA) in an incubator at 37 °C and 5% CO_2_. This assay was conducted according to a modified version of a method described previously^36^. To improve signal detection, cells for the analysis of LPA_1_, LPA_2_, LPA_4_, and LPA_5_ were transfected with Gα-chimeric proteins, whereas those for LPA_4_, LPA_5_, LPA_6_ analysis were treated with the LPA_1–3_ antagonist Ki16425. The cells were co-transfected with vectors-expressing human LPA_1–6_, alkaline phosphatase-tagged TGFα (AP-TGFα). As negative controls, cells transfected with an empty vector instead of the receptor-expressing vector were used. After 24 h, the cells were resuspended in Hank’s balanced salt solution (HBSS), seeded in 96-well plates, and stimulated with cPA analogues administered at tenfold concentration of the final concentration. The cPA analogues were diluted with HBSS, 0.01% protease and fatty acid-free BSA (SERVA Electrophoresis GmbH, Heidelberg, Germany). Cells for LPA_4_, LPA_5_, and LPA_6_ analysis were treated with Ki16425 (final concentration: 10 μM), 5 min before treatment with the cPA analogues. After incubation for 1 h at 37 °C, 80 µL of the supernatant was transferred to fresh 96-well plates. An equal volume of 1 mM *p*-NPP solution [120 mM Tris–HCl (pH 9.5), 40 mM NaCl, and 10 mM MgCl_2_] was added to both the supernatant and the cells. Alkaline phosphatase activity was calculated by measuring absorbance at 405 nm using the Cytation 3 plate reader (BioTek, Winooski, VT, USA).

### Measurement of ATX activity

#### Choline oxidase assay of LPC cleavage

Human recombinant ATX (50 nM final concentration, 5 µL in ATX assay buffer: 50 mM Tris, 140 mM NaCl, 5 mM KCl, 1 mM CaCl_2_, 1 mM MgCl_2_, pH 8.0) and cPA analogues (5 µL each in ATX assay buffer) at various concentrations were added in a 96-well plate and mixed with 90 µL of LPC 16:0 (final concentration: 300 µM) as substrate, 4-aminoantipyrine, TOOS (final concentration: 0.05% w/v each), choline oxidase and peroxidase (final concentration: 10 units/mL each) in ATX assay buffer. The absorbance was measured at 555 nm using the Cytation 3 plate reader (BioTek) at t = 0 and after 2 h of incubation at 37 °C. Inhibition was calculated as a percentage (%) of reduction in absorbance compared to absorbance in the vehicle control (without cPA analogues) after subtraction of the value measured at time 0.

#### LC-QqQ assay of LPC cleavage

ATX (4 µL, final concentration: 50 nM) and cPA analogues (4 µL, final concentration: 10 µM) were added to a 200-µL siliconized microcentrifuge tube and mixed with 32 µL of LPC 16:0 (final concentration: 300 µM) in ATX assay buffer. After incubation at 37 °C for 2 h, 160 µL of acidic methanol was added to stop enzymatic reaction. Sample solutions were filtered, and the LPA 16:0 production was analysed using LC-QqQ as per methods described previously^4^. Inhibition was calculated as a percentage (%) of reduction in LPA production compared to LPA production in the vehicle control (without cPA analogues).

### Docking simulation of cPA analogues and ATX

Initial structure of the human ATX with LPA 18:1 was modelled from the mouse ATX in complex with LPA 18:1 (PDB: 3NKP)^37^ by homology modelling using Prime (Schrödinger, New York, NY, USA). Sequence identity of ATX between human and mouse was 94%. The human ATX structure with LPA 18:1 was refined for docking simulations using the Protein Preparation Wizard Script within Maestro (Schrödinger). For all cPA analogue molecules ionization and energy minimization were performed by the OPLS3e force field in the LigPrep Script of Maestro. These minimized structures were used as input structures for the docking simulations, with performed using the Glide^38,39^ SP docking program (Schrödinger) with a grid box defined by LPA 18:1 molecule and a core constraint using the maximum common substructure with the LPA 18:1. The structural models in the figures were depicted using PyMOL version 2.4 software (Schrödinger).

### LPA, 2carbaLPA, and 3carbaLPA production by ATX

Analyses of LPA from cPA, 2carbaLPA from 2ccPA, and 3carbaLPA from 3ccPA were performed using LC-QqQ as per methods described previously^4^. cPA 18:1, or 2ccPA 18:1 and 3ccPA 18:1 mixture as substrate (10 μM each) were incubated at 37 °C for 2 h with human recombinant ATX (final concentration: 50 nM) and subjected to LC-QqQ. 2carbaLPA and 3carba LPA were both detected in the negative ion mode with selected reaction monitoring (SRM) of the transition from *m/z* 433.27 to *m/z* 151.02. To separate α-LPA and the predicted β-LPA or 2carbaLPA and 3carbaLPA, LC method was performed as per previously described protocols with some modifications^35^. Briefly, the UHPLC PEEK Inertsil ODS-3 C18 column (3 µm, 2.1 × 150 mm; GL Sciences, Tokyo, Japan) was used and the compounds were eluted with a gradient solvent system: solvent A (5 mM ammonium formate in water containing 0.0096% formic acid, pH 4.0), solvent B (5 mM ammonium formate in 95% acetonitrile/5% water containing 1.16% formic acid, pH 4.0). The analytical procedure commenced with solvent B 55% for 10 min, followed by a linear gradient 85% for 40 min that was maintained at 85% for 15 min. Subsequently, solvent B was immediately decreased to the initial condition of 55% and was maintained for 10 min (total 75 min run). The flow rate was 0.2 mL/min at a column temperature of 40 ℃. The injection volume was 10 µL/sample.

### Statistical analysis

All values were analysed using GraphPad Prism 8.4.3 software (GraphPad Software, San Diego, CA, USA) and are reported as means and standard deviation unless otherwise mentioned.

## Supplementary Information

Supplementary Information.

## Data Availability

The data that support the findings of this study are available from the corresponding author upon reasonable request.
