# Supplementary material for: 2-Carba-lysophosphatidic acid is a novel β-lysophosphatidic acid analogue with high potential for lysophosphatidic acid receptor activation and autotaxin inhibition
Source: Sci Rep. 2021 Aug 30;11:17360. doi: 10.1038/s41598-021-96931-2 (PMC8405639; doi:10.1038/s41598-021-96931-2)
Supplement: Supplementary file 1 — Supplementary Information. [file 41598_2021_96931_MOESM1_ESM.docx]

**2-Carba-lysophosphatidic acid is a novel β-lysophosphatidic acid analogue with high potential for lysophosphatidic acid receptor activation and autotaxin inhibition**

**Authors**

Keiko Fukasawa^a,†^, Mari Gotoh^b,†,*^, Akiharu Uwamizu^c,d^, Takatsugu Hirokawa^e,f,g^, Masaki Ishikawa^h^, Yoshibumi Shimizu^a,1^, Shinji Yamamoto^i^, Kensuke Iwasa^i^, Keisuke Yoshikawa^i^, Junken Aoki^c,d^, Kimiko Murakami-Murofushi^a,**^

^†^These authors equally contributed to this study.

**Affiliations**

^a^ Ochadai Academic Production, Ochanomizu University, 2-1-1 Ohtsuka, Bunkyo-ku, Tokyo, 112-8610, Japan.

^b^ Institute for Human Life Innovation, Ochanomizu University, 2-1-1 Ohtsuka, Bunkyo-ku, Tokyo, 112-8610, Japan.

^c^ Department of Health Chemistry, Graduate School of Pharmaceutical Sciences, The University of Tokyo, 7-3-1 Hongo, Bunkyo-ku, Tokyo 113-0033, Japan.

^d^ AMED-LEAP and AMED-CREST, Japan Science and Technology Corporation, 4-1-8 Honcho, Kawaguchi, Saitama 332-0012, Japan.

^e^ Cellular and Molecular Biotechnology Research Institute, National Institute of Advanced Industrial Science and Technology, 2-4-7 Aomi, Koto-ku, Tokyo 135-0064, Japan.

^f^ Transborder Medical Research Center, University of Tsukuba, 1-1-1 Tennodai, Tsukuba, Ibaraki 305-8575, Japan

^g^ Division of Biomedical Science, Faculty of Medicine, University of Tsukuba, 1-1-1 Tennodai, Tsukuba, Ibaraki 305-8575, Japan.

^h^ Clinical Omics Unit, Department of Applied Genomics, Kazusa DNA Research Institute, 2-5-23 Kazusa Kamatari, Kisarazu, Chiba 292-0818, Japan.

^i^ Department of Pharmacology, Faculty of Medicine, Saitama Medical University, 38 Moro-hongo, Moroyama-machi, Iruma-gun, Saitama 350-0495, Japan.

**^*^** Corresponding author at: Institute for human life innovation, Ochanomizu University, 2-1-1 Ohtsuka, Bunkyo-ku, Tokyo 112-8610, Japan.

**^**^**Corresponding author at: Ochadai Academic Production, Ochanomizu University, 2-1-1 Ohtsuka, Bunkyo-ku, Tokyo 112-8610, Japan.

Email-address: [gotoh.mari@ocha.ac.jp](mailto:gotoh.mari@ocha.ac.jp) (M. Gotoh), [murofushi.kimiko@ocha.ac.jp](mailto:murofushi.kimiko@ocha.ac.jp) (K. Murakami-Murofushi).

^1^Present address: Laboratory of Racing Chemistry, 1731-2, Tsurutamachi Utsunomiya, Tochigi 320-0851, Japan.

**Supplementary Figures**

**
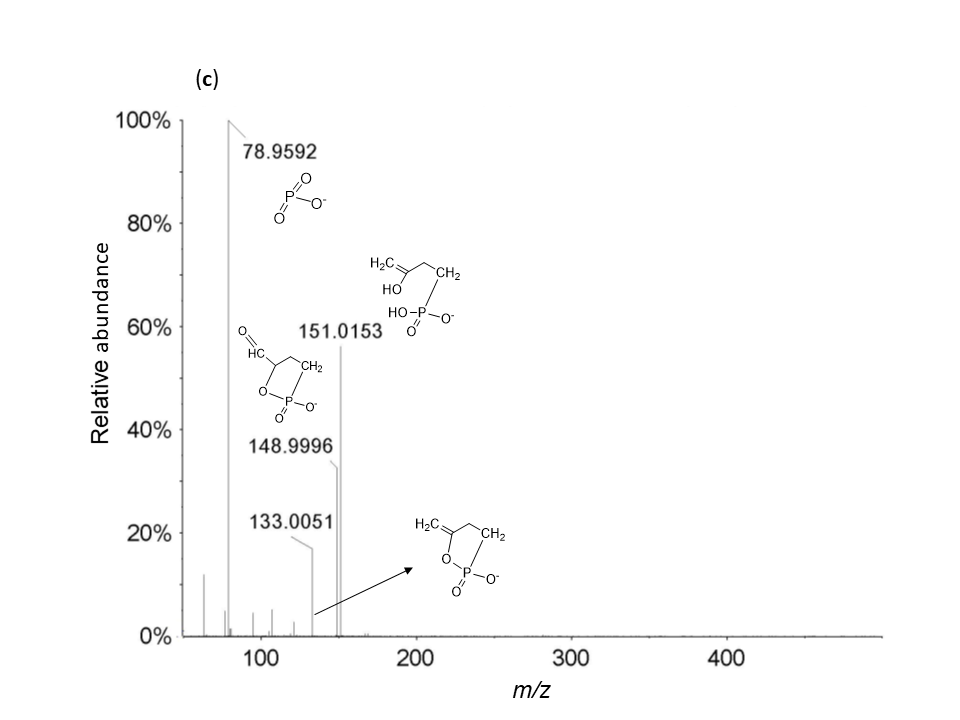

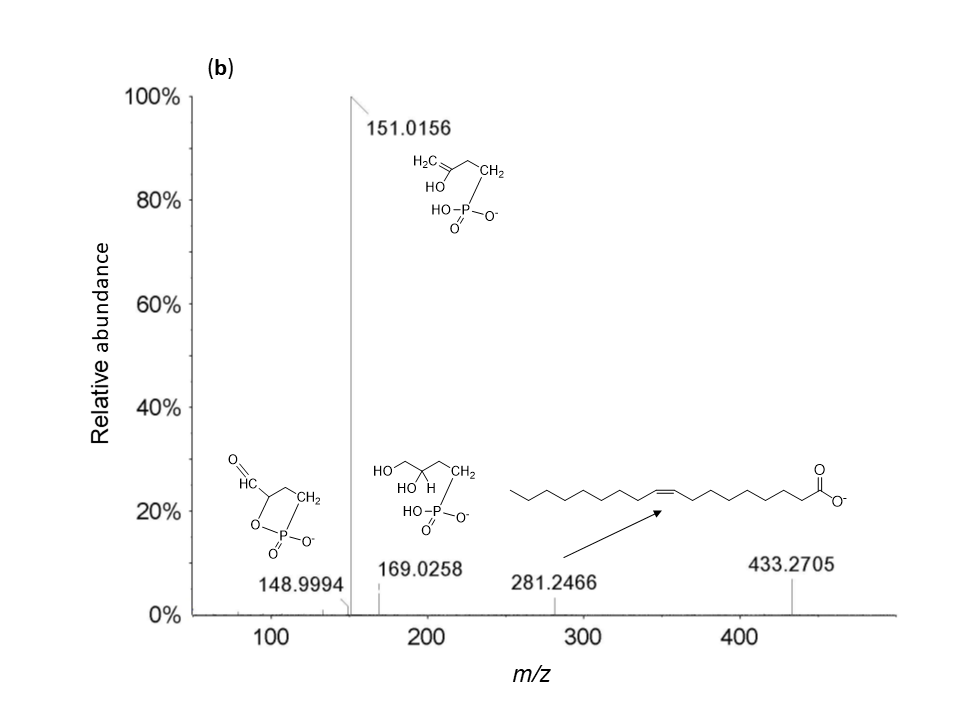

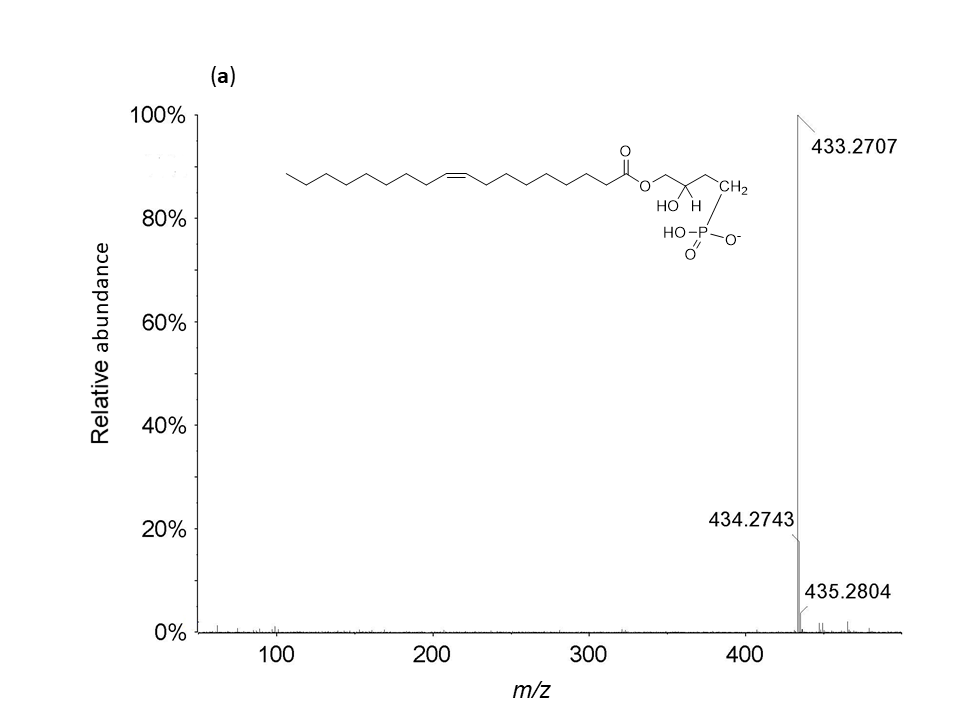
**

**Supplementary Figure S1.** Full mass negative ion scan and proposed chemical structure of (**a**) 3-carba-cyclic phosphatidic acid (3ccPA) degradation compound determined by quadrupole time-of-flight mass spectrometry (QqTOF). (**b**, **c**) Product ion analysis of 3ccPA degradation compound at *m*/*z* 433.2707 performed by QqTOF using a peak width setting of 1.4 Th, by selection of the molecular ion in the first quadrupole and collision activation in the second quadrupole with a collision energy of (**b**) −30 and (**c**) −75 eV. Proposed structures of the product ions observed in the tandem mass spectrometry spectra are shown. Background subtraction was performed using the PeakView software version 1.2 (Sciex).


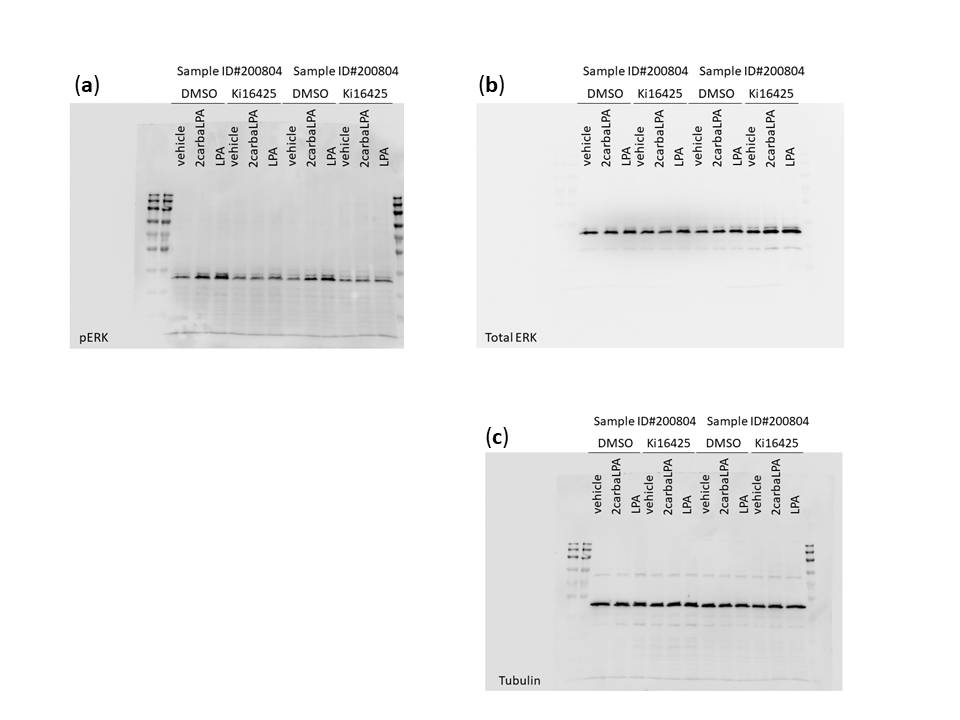


**Supplementary Figure S2.** Effects of 2-carba-lysophosphatidic acid (2carbaLPA) on the phosphorylation of the extracellular signal-regulated kinase (ERK) protein. The full-length blots of Fig. 3 in the main text. HeLa cells were preincubated with 10 µM Ki16425 for 30 min at 37 °C and then incubated with 10 µM 2carbaLPA, lysophosphatidic acid (LPA) 18:1, and vehicle for 5 min at 37 °C. Protein levels of phosphorylated ERK (pERK) (**a**), ERK (**b**), and α-tubulin (**c**) were determined by western blot analysis.

***
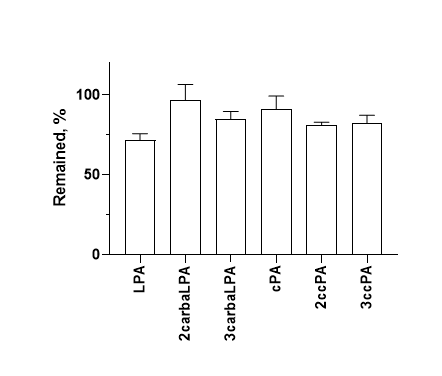
***

**Supplementary Figure S3.** Cyclic phosphatidic acid (cPA) analogue stability in the HEK cell medium. Each cPA analogue (final concentration: 10 μM) was incubated with the HEK cell medium for 1 h at 37 °C. The remaining cPA analogue was quantified using liquid chromatography with tandem mass spectrometry (LC-QqQ) and calculated based on the percentage (%) ratio of the final concentration after incubation to the initial concentration. Data are represented as mean ± standard deviation (n = 3 samples).

**
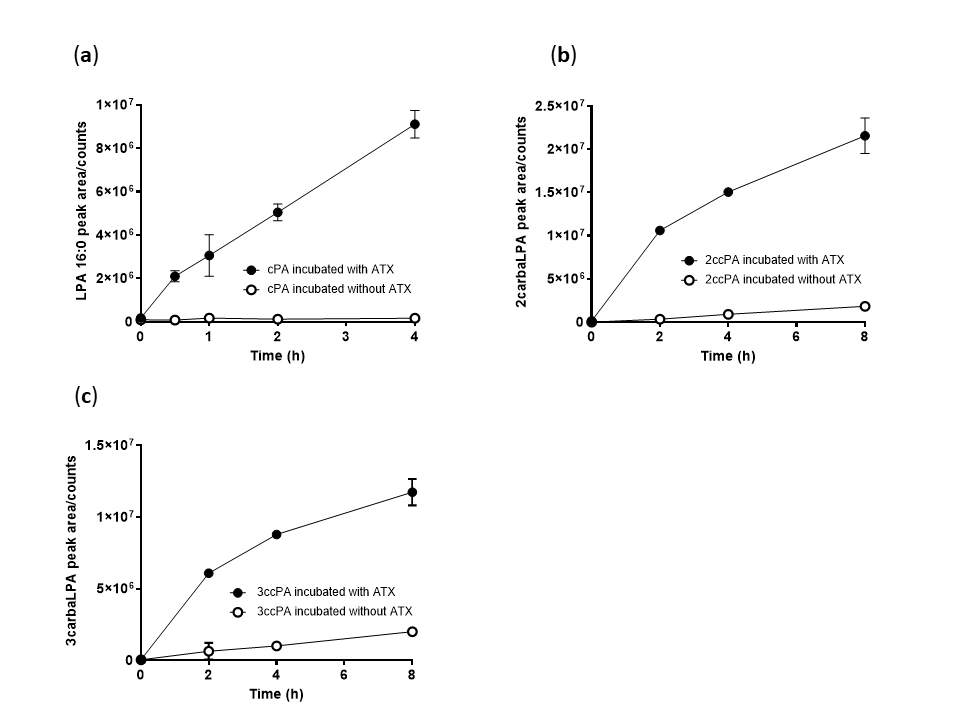
**

**Supplementary Figure S4.** Lysophosphatidic acid (LPA) produced from cyclic phosphatidic acid (cPA), 2-carba-lysophosphatidic acid (2carbaLPA) produced from 2-carba-cyclic phosphatidic acid (2ccPA) and 3-carba-lysophosphatidic acid (3carbaLPA) produced from 3-carba-cyclic phosphatidic acid (3ccPA) by autotaxin (ATX). (**a**) cPA 16:0, (**b**) 2ccPA or (**c**) 3ccPA (final concentration: 10 µM each) was incubated with or without human recombinant ATX (50 nM final concentration) at 37°C. The amount of produced (**a**) LPA 16:0, (**b**) 2carbaLPA or (**c**) 3carbaLPA was detected using liquid chromatography with tandem mass spectrometry (LC-QqQ) and presented as peak area (counts). Data are represented as mean ± standard deviation of experiments conducted in duplicate.


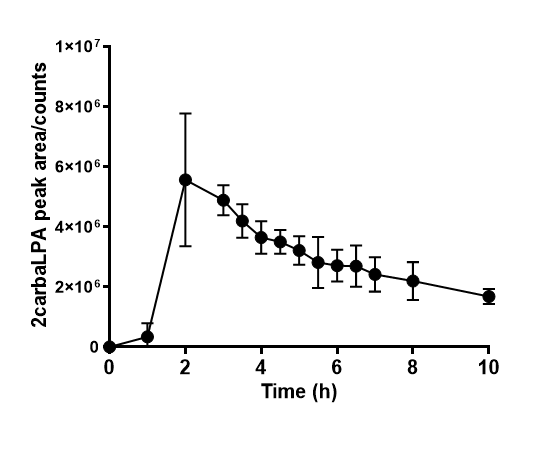


**Supplementary Figure S5.** 2-carba-lysophosphatidic acid (2carbaLPA) produced in rat plasma from rats administered 2-carba-cyclic phosphatidic acid (2ccPA) as a gastro-resistant capsule. Rats were orally administered with 2ccPA containing capsule and 2carbaLPA produced in plasma was detected using liquid chromatography with tandem mass spectrometry (LC-QqQ) and presented as peak area (counts). Data are represented as mean ± standard deviation (n = 5 rat samples).

**Supplementary Materials and Methods**

**cPA analogue stability in the 293A cell medium**

293A cells were prepared in Hank’s balanced salt solution (HBSS) in 96-well plates. Each cPA analogue (LPA 18:1, 2carbaLPA, 3carbaLPA, cPA 18:1, 2ccPA, and 3ccPA) was diluted with HBSS plus 0.01% BSA and was added to the HEK293A cell medium at a final concentration of 10 µM, similar to the TGFα-shedding assay protocol. After incubation for 1 h at 37 ℃, 80 µL of the supernatant was collected and mixed with 320 µL of acidic methanol, including LPA 17:0 and cPA 17:0 (final concentration: 0.2 µM each; Avanti Polar Lipids, Alabaster, AL, USA). After filtration, samples were applied to LC-QqQ and each compound was quantified in the same way as described in the main text. cPA analogues were detected with deprotonated molecular ion [M-H]^−^ (*m*/*z* 435.3 for LPA 18:1, *m*/*z* 433.3 for 2carbaLPA and 3carbaLPA, *m*/*z* 417.2 for cPA 18:1, and *m*/*z* 415.3 for 2ccPA and 3ccPA) as the precursor ion and also detected with fragment ion (*m*/*z* 153.0 for LPA 18:1, *m*/*z* 151.0 for 2carbaLPA and 3carbaLPA, and *m*/*z* 281.2 for cPA 18:1, 2ccPA and 3ccPA) as the product ion.

**LPA, 2carbaLPA, and 3carbaLPA production by ATX**

Analyses of LPA from cPA, 2carbaLPA from 2ccPA, 3carbaLPA from 3ccPA production, with or without ATX, were performed using LC-QqQ as above. cPA 16:0 (Avanti Polar Lipids), 2ccPA or 3ccPA as substrate (10 μM each) were incubated with human recombinant ATX (final concentration: 50 nM) in ATX assay buffer in a 200-µL siliconized microcentrifuge tube. After 37 °C incubation for 0.5, 1, 2, and 4 h for cPA, 2, 4, and 8 h for 2ccPA or 3ccPA, 160 µL of acidic methanol was added in each tube and the mixtures were subjected to LC-QqQ.

**2carbaLPA produced from administered 2ccPA and detection in rat plasma**

Preparation of 2ccPA intended to be administered as a gastro-resistant capsule, administration of the prepared 2ccPA in rats, and extraction of lipids from plasma samples were performed as per previously described methods^1^. The experimental protocols were approved by the Animal Research Committee of the Saitama Medical University (approval number; 2113). Briefly, each capsule was filled with 19.70 ± 1.39 mg of 2ccPA and enteric-coated. Male 7-week-old Sprague Dawley rats were orally administrated with a single capsule and blood was collected from the jugular vein using a catheter from 0 to 10 h. Plasma was obtained by mixing with EDTA-Na_2_ (1 mg/mL), and the lipid fraction was extracted by adding acidic methanol, including LPA 17:0 (final concentration: 100 nM; Avanti Polar Lipids) as internal standard. Analysis for detection of 2carbaLPA with LC-QqQ was performed as above.

**Supplementary References**

1. Shimizu, Y. *et al.* Evaluation of the pharmacokinetics of 2-carba-cyclic phosphatidic acid by liquid chromatography-triple quadrupole mass spectrometry. *Prostaglandins Other Lipid Mediat.* **150**, 106450 (2020).
